# Residual neurotoxicity in ovarian cancer patients in clinical remission after first-line chemotherapy with carboplatin and paclitaxel: The Multicenter Italian Trial in Ovarian cancer (MITO-4) retrospective study

**DOI:** 10.1186/1471-2407-6-5

**Published:** 2006-01-07

**Authors:** Sandro Pignata, Sabino De Placido, Rosalbino Biamonte, Giovanni Scambia, Giovanni Di Vagno, Giuseppe Colucci, Antonio Febbraro, Marco Marinaccio, Alessandra Vernaglia Lombardi, Luigi Manzione, Giacomo Cartenì, Mario Nardi, Saverio Danese, Maria Rosaria Valerio, Andrea de Matteis, Bruno Massidda, Giampietro Gasparini, Massimo Di Maio, Carmela Pisano, Francesco Perrone

**Affiliations:** 1 Medical Oncology B, National Cancer Institute, Naples, Italy; 2 Department of Molecular and Clinical Endocrinology and Oncology, University Federico II, Naples, Italy; 3 Medical Oncology, Mariano Santo Hospital, Cosenza, Italy; 4 Gynecologic Oncology Unit, Catholic University of Rome, Rome, Italy; 5 Obstetrics II, Department of Obstetrics and Gynecology, University of Bari, Bari, Italy; 6 Medical and Experimental Oncology Unit, Oncology Institute, Bari, Italy; 7 Medical Oncology, Fatebenefratelli Hospital, Benevento, Italy; 8 Obstetrics I, Department of Obstetrics and Gynecology, University of Bari, Bari, Italy; 9 Malzoni Clinic, Avellino, Italy; 10 Medical Oncology, San Carlo Hospital, Potenza, Italy; 11 Medical Oncology, Cardarelli Hospital, Naples, Italy; 12 Division of Medical Oncology, Ospedali Riuniti, Reggio Calabria, Italy; 13 Ginecology, S Anna Hospital, Turin, Italy; 14 Medical Oncology, University of Palermo, Palermo, Italy; 15 Medical Oncology C, National Cancer Institute, Naples, Italy; 16 Medical Oncology, University of Cagliari, Cagliari, Italy; 17 Division of Medical Oncology, San Filippo Neri Hospital, Rome, Italy; 18 Clinical Trials Unit, National Cancer Institute, Naples, Italy

## Abstract

**Background:**

Carboplatin/paclitaxel is the chemotherapy of choice for advanced ovarian cancer, both in first line and in platinum-sensitive recurrence. Although a significant proportion of patients have some neurotoxicity during treatment, the long-term outcome of chemotherapy-induced neuropathy has been scantly studied. We retrospectively assessed the prevalence of residual neuropathy in a cohort of patients in clinical remission after first-line carboplatin/paclitaxel for advanced ovarian cancer.

**Methods:**

120 patients have been included in this study (101 participating in a multicentre phase III trial evaluating the efficacy of consolidation treatment with topotecan, and 19 treated at the National Cancer Institute of Naples after the end of the trial). All patients received carboplatin (AUC 5) plus paclitaxel (175 mg/m^2^) every 3 weeks for 6 cycles, completing treatment between 1998 and 2003. Data were collected between May and September 2004. Residual sensory and motor neurotoxicity were coded according to the National Cancer Institute – Common Toxicity Criteria.

**Results:**

55 patients (46%) did not experience any grade of neurological toxicity during chemotherapy and of these none had signs of neuropathy during follow-up. The other 65 patients (54%) had chemotherapy-induced neurotoxicity during treatment and follow-up data are available for 60 of them. Fourteen out of 60 patients (23%) referred residual neuropathy at the most recent follow-up visit, after a median follow up of 18 months (range, 7–58 months): 12 patients had grade 1 and 2 patients grade 2 peripheral sensory neuropathy; 3 patients also had grade 1 motor neuropathy. The remaining 46/60 patients (77%) had no residual neuropathy at the moment of interview: recovery from neurotoxicity had occurred in the first 2 months after the end of chemotherapy in 22 (37%), between 2 and 6 months in 15 (25%), or after more than 6 months in 9 patients (15%). Considering all 120 treated patients, there was a 15% probability of persistent neurological toxicity 6 months after the end of chemotherapy.

**Conclusion:**

A significant proportion of patients with advanced ovarian cancer treated with first-line carboplatin/paclitaxel suffer long-term residual neuropathy. This issue should be carefully taken into account before considering re-treatment with the same agents in sensitive recurrent disease.

## Background

The combination of carboplatin and paclitaxel is considered worldwide the standard first-line chemotherapy for patients with advanced ovarian cancer [1-3]. This chemotherapy is highly effective, and about half of the patients show a clinical complete remission after six cycles [1]. Unfortunately, most of these patients experience disease recurrence. A significant proportion of recurring patients, with a platinum-free interval longer than 6–12 months, are classified as platinum-sensitive and are candidates for re-treatment with the same drugs used in first-line [4]. However, the use of carboplatin and paclitaxel to treat recurrent disease may be limited by the risk of cumulative peripheral neurotoxicity, a common side effect of this combination when given as first-line chemotherapy in ovarian cancer. Grade 2–3 sensory toxicity, according to the National Cancer Institute Common Toxicity Criteria [5] is reported in about 25% of the patients, and also motor toxicity can be experienced, although less frequently [1-3,6,7]. The most common feature of carboplatin/paclitaxel neurotoxicity is sensory distal neuropathy [8]. A mixture of paresthesias and dysesthesias is often prominent, and complaints include burning dysesthesia, numbness, tingling and shooting, typically in a stocking-glove distribution. The peripheral neuropathy related to carboplatin/paclitaxel is due to axonopathy, while motor and autonomic nerves appear to be less affected. Mild weakness is the most common complaint of motor toxicity, generally due to distal motor neuropathy [8].

The occurrence of neurotoxicity may have significant effects on quality of life, and several reliable instruments containing items specifically addressing symptoms related to neurotoxicity have been developed [9,10]. These instruments have been used in randomized studies comparing cisplatin/paclitaxel versus carboplatin/paclitaxel [1] and docetaxel/carboplatin versus paclitaxel/carboplatin [7].

Despite the attention dedicated to neurotoxicity, the long-term outcome of peripheral neuropathy induced by carboplatin/paclitaxel has not been deeply investigated. In particular, the proportion of platinum-sensitive recurring patients who are candidates for retreatment with the same regimen, but who are at increased risk of cumulative toxicity because of residual neuropathy, has not been evaluated.

In this study, we retrospectively evaluated the prevalence of residual neuropathy in a cohort of ovarian cancer patients in clinical remission following first-line chemotherapy with carboplatin and paclitaxel.

## Methods

Patients included in this analysis received first-line chemotherapy for advanced ovarian cancer between 1998 and 2003. Most of the patients participated in the Multicenter Italian Trials in Ovarian cancer (MITO)-1 study [11], that was a randomized phase III trial that evaluated the efficacy of consolidation treatment with topotecan in patients responding to first-line chemotherapy with carboplatin and paclitaxel. At the moment of the present retrospective study (May–September 2004), 101 out of the 273 patients enrolled in the MITO-1 study were in clinical remission and were eligible for the present analysis. Among these patients, 41 had received 4 cycles of topotecan, at the dose of 1.5 mg/sm from day 1 to 5 every 3 weeks, after completion of the 6 cycles of carboplatin and paclitaxel, according to the MITO-1 protocol; these patients were not excluded from this analysis because topotecan is not expected to produce significant neurotoxicity. A further group of 19 patients, among those treated at the National Cancer Institute of Naples after the end of the MITO-1 accrual, were in clinical remission at the moment of this study and were eligible for this retrospective study. For all of the 120 patients described above, primary treatment was carboplatin – area under the curve (AUC) 5 – and paclitaxel – 175 mg/sm given in a 3-hour infusion.

This was designed as a retrospective study, so specific approval by the Ethics Committee was not required by the Italian law. Of course, all the patients participating in the MITO-1 trial gave a written informed consent before any study procedure. The other 19 patients received first-line treatment with carboplatin and paclitaxel as part of the standard clinical practice at the National Cancer Institute of Naples.

Information about neurotoxicity experienced by the patients during the treatment was collected from the database of the MITO-1 trial for 101 patients, and from clinical files for the remaining 19 patients treated outside the trial. Residual neurotoxicity was evaluated by the physician and graded according to the NCI-CTC criteria, version 2.0 [5]. According to these criteria, sensory neuropathy is coded as grade 1 (loss of deep tendon reflexes or paresthesia, including tingling, but not interfering with function), grade 2 (objective sensory loss or paresthesia, including tingling, interfering with function, but not interfering with activities of daily living), grade 3 (sensory loss or paresthesia interfering with activities of daily living) or grade 4 (permanent sensory loss that interferes with function). Motor neuropathy is coded as grade 1 (subjective weakness but no objective findings), grade 2 (mild objective weakness interfering with function, but not interfering with activities of daily living), grade 3 (objective weakness interfering with activities of daily living) or grade 4 (paralysis).

Follow-up data were collected between May and September 2004. After performing a clinical examination and an interview, the participating investigators completed a dedicated case report form for each patient, reporting the grade of any residual sensory and/or motor neurotoxicity. Details of pharmacological treatments administered for neuropathy were also collected. For those patients who had experienced neurotoxicity during chemotherapy but had no residual neuropathy at the moment of the interview, the investigator reported the date of resolution of neuropathy, as referred by the patient.

Time to resolution of neuro-toxicity was defined as the interval from the end of chemotherapy with carboplatin and paclitaxel to the date of resolution of neuro-toxicity or the date of last follow-up information for patients with residual neuro-toxicity. Time to resolution curve was drawn with the Kaplan-Meier product limit method [12].

Analyses were performed with S-PLUS software (S-PLUS 6.0 Professional, release 1; Insightful Corporation, Seattle, WA, USA).

## Results

The median age of the 120 patients included in the study was 56 years (range, 26–72). The median follow-up, defined as the interval between the end of chemotherapy and the date of the interview, was 48 months (range 7–74).

Table 1 shows the details of neurotoxicity recorded during treatment with carboplatin and paclitaxel, and the details of residual neuropathy. Fifty-five patients (46%) did not suffer neurological toxicity during chemotherapy, and none of these patients had signs of neuropathy at the moment of the interview. The remaining 65 patients (54%) suffered neurological toxicity during chemotherapy. In particular, 51 patients (42%) experienced grade 1 sensory neuropathy, 13 (11%) grade 2 sensory neuropathy and 1 (1%) grade 3 sensory neuropathy. Three patients experienced motor deficit in addition to sensory neuropathy.

**Figure 1:**
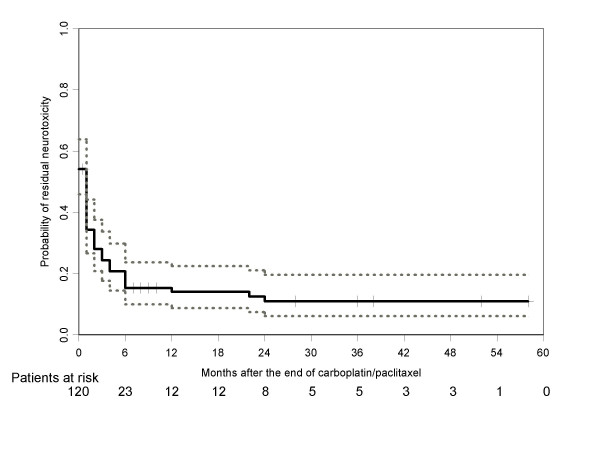
Kaplan-Meyer curve of time to resolution of neurological toxicity in patients with epithelial ovarian carcinoma treated with the combination of carboplatin and paclitaxel (dotted lines represent 95% confidence interval).

**Figure 2:**
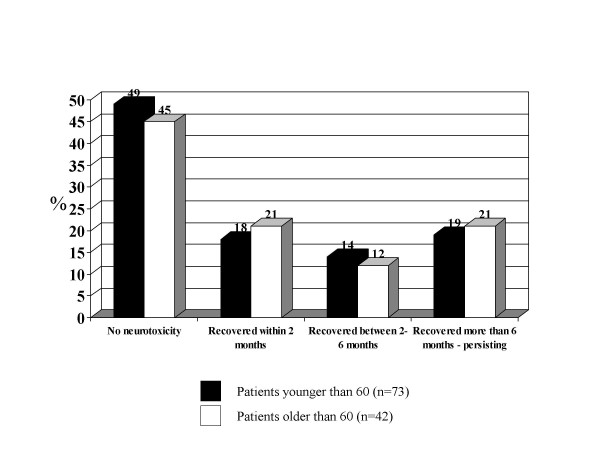
Duration of residual neuro-toxicity according to age category.

Follow-up data are available for 60 out of the 65 patients who experienced neurotoxicity during chemotherapy. Forty-six patients (77%) reported resolution of neuropathy during follow-up and had no residual neuropathy at the moment of interview. Complete recovery occurred in the first 2 months after the end of chemotherapy in 22 patients (37%), but 15 patients (25%) recovered from neuropathy between 2 and 6 months, and 9 patients (15%) after 6 months or more. The remaining 14 out of 60 cases (23%) had some grade of residual neuropathy at the moment of assessment (median follow-up 23 months, range 7–58). Most of these patients suffered from grade 1 peripheral sensory neuropathy (12 patients), but 2 patients had grade 2 sensory neuropathy and 3 patients reported complaints derived by a grade 1 motor neuropathy in addition to sensory neurotoxicity. Considering all 120 patients who received combination chemotherapy with carboplatin and paclitaxel, the probability of neurological toxicity for a patient was 54% during chemotherapy, 15% at 6 months after the end of chemotherapy, 14% at 1 year after the end of chemotherapy and 11% at 2 years after the end of chemotherapy (Figure 1). No significant difference (p = 0.94) was found in the duration of neurological toxicity when patients were divided in two cohorts according to age, younger or older than 60 (Figure 2).

**Figure 3:**
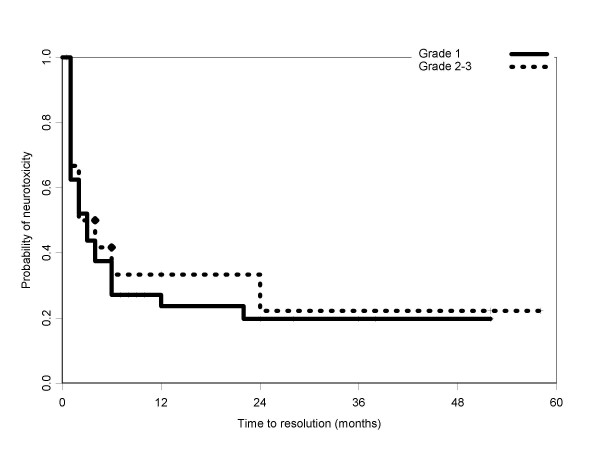
Kaplan-Meyer curve of time to resolution of neurological toxicity according to grade of toxicity reported during chemotherapy.

Figure 3 shows the time to resolution of neurotoxicity according to its severity. Six-months probability of residual neuropathy was 27.1% for patients suffering from grade 1 neurotoxicity at the end of chemotherapy and 33.3% for patients with grade 2 or more. 1-year probability of residual neuropathy was 23.7% and 33.3%, and 2-year probability was 19.7% and 22.2%, for patients with grade 1 and grade 2 or more, respectively. Difference of time to resolution among the two groups of patients was not statistically significant (p = 0.716, Log-rank test).

No specific pharmacological therapy for neurotoxicity was delivered, with the exception of one patient who received corticosteroids and gabapentin.

## Discussion

In the present retrospective analysis, we explored the prevalence of residual neuropathy in a cohort of patients with advanced ovarian cancer in complete remission after first-line chemotherapy. Our data show that a significant proportion of patients who experience neurological toxicity during treatment with carboplatin and paclitaxel suffer from prolonged residual neuropathy during their progression-free follow-up.

Three randomized studies have compared carboplatin/paclitaxel versus cisplatin/paclitaxel as first-line chemotherapy in advanced ovarian cancer [1-3]. All these studies showed that carboplatin/paclitaxel induces lower neurotoxicity, with a significant positive impact on quality of life. Based on these results, the combination of carboplatin and paclitaxel is now considered the chemotherapy of choice for ovarian cancer. Nevertheless, about 40% of patients treated with carboplatin and paclitaxel experience grade 1, 21% grade 2 and 6% grade 3 neurotoxicity during treatment [1-3]. The presence of residual neuropathy is an even more important issue after the publication of the ICON-4 study, that showed a survival benefit for platinum-sensitive recurrent patients treated with carboplatin/paclitaxel combination, compared to carboplatin alone [4]. The persistence of residual neuropathy could increase the risk of cumulative toxicity, and limit the use of carboplatin/paclitaxel in the recurrence setting. Furthermore, the debated maintenance strategy, with extended administration of paclitaxel after complete response to first-line chemotherapy, supported by the study of Markman et al. [13], can be seriously limited by the persistence of residual neuropathy.

In our analysis after a median follow-up of 48 months, while none of the patients without neurotoxicity during first-line had signs of neuropathy during follow-up, 23% of patients with chemotherapy-induced neurological toxicity had residual neuropathy. Neuropathy was in most cases sensory, but three cases also had residual weakness related to motor damage. Furthermore, within the group of patients without residual neuropathy at the time of study assessment, a significant proportion had recovery from neuropathy more than 6 months after the end of treatment. It should be noted that probability of long-term persistence of neurotoxicity was not negligible either for patients experiencing moderate to severe toxicity during chemotherapy, or those patients suffering from grade 1 toxicity.

These data suggest that residual neuropathy could affect a proportion of patients higher than believed. Since the patients that experience recurrent disease after more than 6 months from the end of chemotherapy are candidates for re-treatment with carboplatin/paclitaxel [4], it seems important to recommend an accurate assessment of the presence of residual neuropathy before starting re-treatment. Recent data show that treatment with paclitaxel may induce cumulative neurotoxicity in patients with neuropathy during a previous cisplatin-based chemotherapy [14]. The results of the ICON-4 study show that re-treatment with carboplatin and paclitaxel is associated with neurotoxicity grade 2–3 in up to 20% of the patients [4].

Unfortunately, the retrospective nature of our study did not allow an evaluation of the effects of neuropathy on health-related quality of life. This may be an important issue particularly for early stage ovarian cancer patients treated with carboplatin/paclitaxel adjuvant therapy. In the phase III randomized trial performed by the Scottish Gynaecological Cancer Trials Group, comparing the combination of carboplatin and docetaxel with the standard carboplatin/paclitaxel combination, health-related quality of life was one of the secondary end-points of the study [7]. Coherently with the higher incidence of neurotoxicity in the carboplatin/paclitaxel arm, quality of life scores related to neurotoxicity deteriorated more in this arm, and patients treated with carboplatin/paclitaxel reported significantly worse scores for acute, persistent and long-term (6-months after treatment) neurotoxicity. These data, together with our results, emphasize that the risk of residual long-term neuropathy should be weighted with the potential benefit of adding paclitaxel to carboplatin in the re-treatment of these patients.

In our opinion, research in the field of prevention of neurological toxicity is mandatory. Some pharmacological attempts have been done with chemoprotective agents, administered with the aim of minimizing chemotherapy-induced neurotoxicity. Conflicting data have been published on the effect of amifostine in preventing carboplatin/paclitaxel neurotoxicity. Although "in vitro" data indicate that amifostine can prevent neurotoxicity [15], clinical data are not so clear [16,17]. Some findings suggest that acetyl-L-carnitine can have a protective role against paclitaxel/cisplatin neurotoxicity [18]. However, as confirmed by the finding that only one patient in our study received a pharmacological treatment for neurotoxicity, none of these drugs are used in clinical practice.

## Conclusion

Our data indicate that a significant proportion of patients treated with first-line chemotherapy with carboplatin and paclitaxel have long-term residual neuropathy. This issue should be carefully taken into account before considering a re-treatment with carboplatin and paclitaxel in sensitive recurrent disease.

## Competing interests

The author(s) declare that they have no competing interests.

## Authors' contributions

SP, MDM and FP conceived of the study, participated in its design and coordination and drafted the manuscript;

SP, SDP, RB, GS, GDV, GC, AF, AVL, LM, MN, SD, MRV, GG and CP treated the patients, collected the clinical data useful for the analysis and revised the article critically for important intellectual content;

MDM and FP performed the statistical analysis of the data.

All authors read and approved the final manuscript.

## Participating institutions and coauthors

National Cancer Institute, Naples (Medical Oncology B: S Pignata, C Pisano, R Tambaro, E Ferrari, RV Iaffaioli; Ginecology: S Greggi; Medical Oncology C: A de Matteis; Clinical Trials Unit: F Perrone, M Di Maio, E De Maio, A Morabito); Department of Molecular and Clinical Endocrinology and Oncology, University Fererico II, Naples (S De Placido, R Lauria, M De Laurentiis, L Zinno); Medical Oncology, Mariano Santo Hospital, Cosenza (R Biamonte, S Palazzo, A Rovito, A Filice); Gynecologic Oncology Unit, Catholic University of Rome, Rome (G Scambia, D Lorusso, G Ferrandina, A Testa); Obstetrics II, Department of Obstetrics and Gynecology, University of Bari, Bari (G Di Vagno, V Loizzi, L Selvaggi); Medical and Experimental Oncology Unit, Oncology Institute, Bari (G Colucci, E Naglieri, E Maiello); Medical Oncology, Fatebenefratelli Hospital, Benevento (A Febbraro, A Campagna, T Pedicini); Obstetrics I, Department of Obstetrics and Ginecology, University of Bari, Bari (M Marinaccio, S Shonauer); Malzoni Clinic, Avellino (A Vernaglia Lombardi, C Malzoni); Medical Oncology, San Carlo Hospital, Potenza (L Manzione, R Romano); Medical Oncology, Cardarelli Hospital, Naples (G Cartenì, T Guida); Division of Medical Oncology, Ospedali Riuniti, Reggio Calabria (M Nardi, P Del Medico); Ginecology, S Anna Hospital, Turin (S Danese, G Richiardi); Medical Oncology, University of Palermo, Palermo (MR Valerio); Division of Medical Oncology, San Filippo Neri Hospital, Rome (G Gasparini); Medical Oncology, Santo Spirito Hospital, Pescara (D Natale); Medical Oncology, University of Cagliari, Cagliari (B Massidda).

## Pre-publication history

The pre-publication history for this paper can be accessed here:



## Figures and Tables

**Table 1:** Characteristics of neurological toxicity during or after first-line chemotherapy with carboplatin plus paclitaxel

**Neurological toxicity**	**No. of patients**	**%**
***A. during chemotherapy (n = 120)***		
**No toxicity**	**55**	**(46%)**
**At least grade 1 toxicity**	**65**	**(54%)**
Type of toxicity		
Sensory, grade 1	51	(42%)
Sensory, grade 2	13	(11%)
Sensory, grade 3	1	(1%)
Motor, grade 1 (combined with sensory)	3	(3%)

***B. residual toxicity after chemotherapy (n = 60 - 5 patients lost)***		
**No toxicity at the moment of interview**	**46**	**(77%)**
Time to recovery from residual neuropathy		
< 2 months after the end of chemotherapy	22	37%
≥ 2 < 6 months after the end of chemotherapy	15	25%
≥ 6 months after the end of chemotherapy	9	15%
**At least grade 1 residual toxicity at the moment of interview**	**14**	**(23%)**
Type of residual toxicity		
Sensory, grade 1	12	(20%)
Sensory, grade 2	2	(3%)
Motor, grade 1 (combined with sensory)	3	(5%)
